# *In* *Situ* Anodization of WO_3_-Decorated TiO_2_ Nanotube Arrays for Efficient Mercury Removal

**DOI:** 10.3390/ma8095270

**Published:** 2015-08-28

**Authors:** Wai Hong Lee, Chin Wei Lai, Sharifah Bee Abd Hamid

**Affiliations:** Nanotechnology & Catalysis Research Centre (NANOCAT), Institute of Postgraduate Studies (IPS), University of Malaya, Kuala Lumpur 50603, Malaysia; E-Mails: leewaihong@siswa.um.edu.my (W.H.L.); sharifahbee@um.edu.my (S.B.A.H.)

**Keywords:** WO_3_ decorated TiO_2_ nanotubes, electrochemical anodization, mercury removal, fluoride content, active surface area

## Abstract

WO_3_-decorated TiO_2_ nanotube arrays were successfully synthesized using an *in situ* anodization method in ethylene glycol electrolyte with dissolved H_2_O_2_ and ammonium fluoride in amounts ranging from 0 to 0.5 wt %. Anodization was carried out at a voltage of 40 V for a duration of 60 min. By using the less stable tungsten as the cathode material instead of the conventionally used platinum electrode, tungsten will form dissolved ions (W^6+^) in the electrolyte which will then move toward the titanium foil and form a coherent deposit on the titanium foil. The fluoride ion content was controlled to determine the optimum chemical dissolution rate of TiO_2_ during anodization to produce a uniform nanotubular structure of TiO_2_ film. Nanotube arrays were then characterized using FESEM, EDAX, XRD, as well as Raman spectroscopy. Based on the FESEM images obtained, nanotube arrays with an average pore diameter of up to 65 nm and a length of 1.8 µm were produced. The tungsten element in the samples was confirmed by EDAX results which showed varying tungsten content from 0.22 to 2.30 at%. XRD and Raman results showed the anatase phase of TiO_2_ after calcination at 400 °C for 4 h in air atmosphere. The mercury removal efficiency of the nanotube arrays was investigated by photoirradiating samples dipped in mercury chloride solution with TUV (Tube ultraviolet) 96W UV-B Germicidal light. The nanotubes with the highest aspect ratio (15.9) and geometric surface area factor (92.0) exhibited the best mercury removal performance due to a larger active surface area, which enables more Hg^2+^ to adsorb onto the catalyst surface to undergo reduction to Hg^0^. The incorporation of WO_3_ species onto TiO_2_ nanotubes also improved the mercury removal performance due to improved charge separation and decreased charge carrier recombination because of the charge transfer from the conduction band of TiO_2_ to the conduction band of WO_3_.

## 1. Introduction

Mercury is one of the earliest known metals and has been used by humankind for more than 2300 years [1,2]. Due to its unique physio-chemical properties (liquid form at STP (standard temperature and pressure), high surface tension, high specific gravity, low electrical resistance), mercury is widely utilized in many industries such as metallurgy, manufacturing, medicine, and mining [1,3]. However, mercury is highly toxic, even at very low concentrations, and its ability to bioaccumulate in the food chain makes mercury pollution a huge threat to the environment and humankind [2,3]. Human exposure to mercury is mainly due to consumption of mercury-contaminated food and occupational exposure [3,4]. Exposure to mercury is hazardous and can cause neurological damage as well as impairment of nerves, muscles, and organs such as kidneys [3,5]. Hg(II) is the most common form of inorganic mercury in the aquatic environment and can be converted into more toxic organic forms through biological methylation [6]. Thus, it is important to develop technologies for the efficient removal of Hg(II) from water. Mercury can be successfully removed from a high concentration solution by membrane filtration, precipitation, ion exchange, and other methods. However, these methods are less efficient and are expensive to use on mercury concentrations lower than 100 ppm. Therefore, for low concentrations of mercury, adsorption techniques are preferred [2,7]. Activated carbon is the most effective adsorbent for mercury removal but it is too expensive for large-scale treatment [7]. This has given rise to the need for alternative adsorbents for mercury decontamination.

Titanium dioxide (TiO_2_) nanotubes have been proven to be a potential adsorbent for many contaminants such as dyes and heavy metals [8,9]. The reason is mainly attributed to TiO_2_ nanotubes having large a specific surface area which enables more pollutant particles to be adsorbed onto the nanotubes. Furthermore, TiO_2_ is a transition metal oxide semiconductor (with a band gap of 3.20 eV) which is active under UV light irradiation. Thus, TiO_2_ can also act as a photoreductor of heavy metal ions when irradiated with UV light. Additionally, the unique features of TiO_2_, such as non-toxicity, cost effectiveness, long-term stability, widespread availability, corrosion stability, and high photocatalytic ability, will complement its effectiveness in this application. However, a major limitation of TiO_2_ is its large band gap of 3.20 eV which only allows it to utilize about 2%–3% of the solar light that reaches the earth [10].

In this present study, tungsten trioxide (WO_3_) was doped onto the TiO_2_ nanotubes using an *in situ* anodization method in order to improve the photocatalytic ability of TiO_2_. WO_3_ with a smaller band gap of 2.3–2.8 eV is active in the visible range. Furthermore, the upper edge of the valence band and the lower edge of the conduction band are lower for WO_3_ than for TiO_2_, thus creating a potential gradient at the composite interface which allows electron transfer from the conduction band of TiO_2_ down to the conduction band of WO_3_. This will improve charge separation and inhibit charge carrier recombination. The influence of fluoride ion content on the growth of WO_3_-TiO_2_ nanotubes was studied, which is important in tailoring the desired length, pore size, and wall thickness of the nanotubes for a high aspect ratio (length/pore size) to achieve effective mercury removal.

## 2. Results and Discussion

### 2.1. Morphological Studies and Elemental Analysis

**Figure 1:**
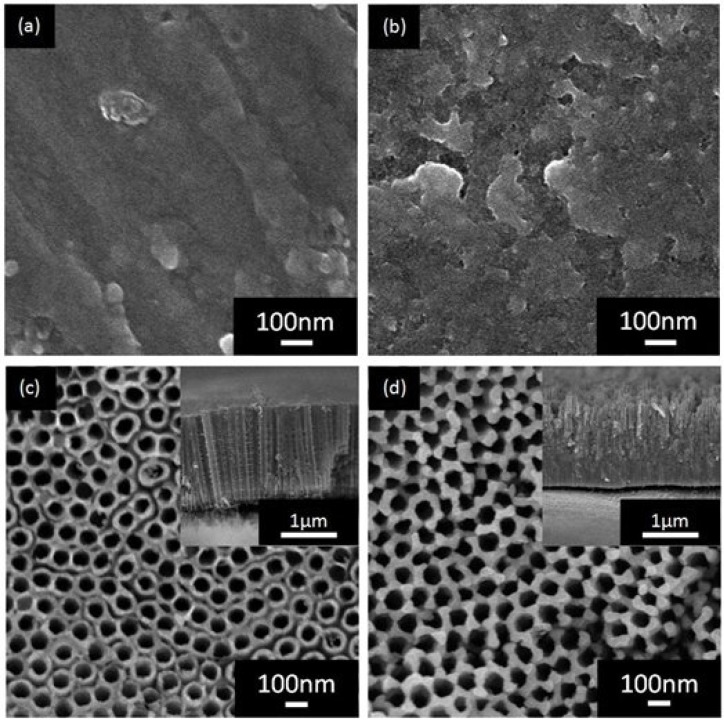
FESEM images of WO_3_-TiO_2_ nanostructures obtained with varying fluoride ion content: (**a**) 0 wt %, (**b**) 0.1 wt %, (**c**) 0.3 wt %, and (**d**) 0.5 wt %. Insets are the side views of the samples.

The effect of fluoride ion content on the morphology of WO_3_-decorated TiO_2_ nanostructure was investigated. Figure 1 shows the surface morphologies of WO_3_-decorated TiO_2_ layers formed using different fluoride ion content from 0 to 0.5 wt %. Fluoride content of 0 wt % produced no nanostructures as presented in Figure 1a, where only an oxide layer of TiO_2_ was observed from the FESEM micrograph. The reason might be attributed to the absence of F^−^ ionic species during the electrochemical anodization stage. As a matter of fact, the high etching power of F^−^ ions plays an important role in chemical etching and dissolution of the TiO_2_ layer to form a porous structure [11,12,13]. Figure 1b shows the FESEM micrograph of the sample prepared in ethylene glycol containing 0.1 wt % NH_4_F. The surface contained irregular features and small oxide pits were observed. The inadequate F^−^ levels probably caused incomplete chemical dissolution and oxidation at the interface between Ti and the barrier layer. For the 0.3 wt % NH_4_F content, a hollow cylindrical oxide was observed, which indicates that the amount of F^−^ present in the ethylene glycol was sufficient to increase the chemical dissolution. This led to further acidification to develop a nanotubular structure, as shown in Figure 1c. The WO_3_-decorated TiO_2_ nanotube arrays with a diameter of approximately 65 nm, length of 1.8 µm, and wall thickness of 24 nm were formed when the F^−^ concentration was increased to 0.3 wt%. When fluoride ion content was increased to 0.5 wt %, the pore diameter increased to approximately 80 nm, but the length and wall thickness decreased to approximately 1.5 µm and 17 nm, respectively. Thus, the optimization of fluoride ion content in order to grow well-aligned WO_3_-decorated TiO_2_ nanotubes was identified to be 0.3 wt % for 60 min of anodization duration at a potential of 40 V. The average diameter, length, and aspect ratio of the nanotubes are summarized in Table 1. The average diameter, length, wall thickness, aspect ratio (AR), and geometric surface area factor (*G*) of the nanotubes formed with varying fluoride ion content are summarized in Table 1. The aspect ratio and geometric area factor were calculated as follows:

(1) AR = *L*/(*D* + 2*w*)

(2) G = [4π*L* (*D* + *w*)]/[√3 (*D* + 2*w*) × 2] + 1

where: L = nanotube length in nm; *D* = pore size; *w* = wall thickness.

**Table 1:** Pore diameter, length, wall thickness, aspect ratio, and geometric surface area factor of WO_3_-TiO_2_ nanotubes formed with varying fluoride ion content.

NH_4_F (wt%)	Diameter (nm)	Length (µm)	Wall Thickness (nm)	AR	*G*
0	–	–	–	–	–
0.1	–	–	–	–	–
0.3	65	1.8	24	15.9	92.0
0.5	80	1.5	17	13.2	82.2

**Figure 2:**
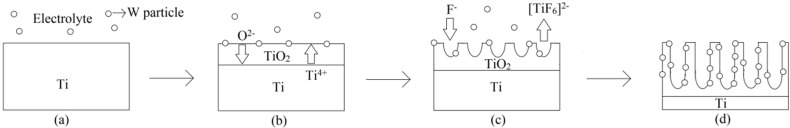
Formation of WO_3_-loaded TiO_2_ nanotubes: (**a**) Ti foil, (**b**) oxide layer formation, (**c**) chemical dissolution of oxide layer, and (**d**) WO_3_-loaded TiO_2_ nanotubes.

As shown in Figure 2, during the initial stage of anodization, field-assisted oxidation occurs on the Ti metal surface, which forms a compact oxide layer. The reaction is represented by the equation below:

(3) Ti^2+^ + H_2_O_2_→TiO_2_ + 2H^+^

Fine pits or cracks then form on the oxide surfaces which arise from chemical and field-assisted dissolution of the oxide at local points of high energy. As further chemical and field-assisted dissolution of the oxide layer occurs, the porous structures transition into nanotube structures and the nanotube array continues to increase in length. The reaction that occurred is represented by the equation below:

(4) TiO_2_ + 4H^+^ + 6F^−^→[TiF_6_]^2−^ + 2H_2_O

The equation below represents the formation of WO_3_ species for the synthesis of the anodic WO_3_-loaded TiO_2_ nanostructure:

(5) W^6+^ + 3H_2_O→WO_3_ + 6H^+^

The quantitative elemental analysis of WO_3_-loaded TiO_2_ nanotubes was carried out by cross-sectional FESEM-EDAX to confirm the presence of W throughout the length of the nanotubes and the average elemental compositions (at %) were obtained by taking eight spots along the nanotubes. The percentage of each element is shown in Table 2. The WO_3_-TiO_2_ nanotubes show the presence of Ti, O, W, and C elements. The sample produced with 0.5 wt % NH_4_F shows the highest at% of W, which is 2.90 at %. The samples produced with 0.3 wt % NH_4_F and 0.1 wt % NH_4_F showed 2.36 at % and 1.50 at % of W, respectively. The sample produced with 0 wt % NH_4_F showed the lowest at % of W, which is 0.22 at %. The presence of W within the nanotube arrays was found to increase with increasing fluoride ion content. This is because increasing fluoride ion content will increase the chemical dissolution rate of W in the electrolyte solution due to the high etching power of F^−^ ions, thereby increasing the amount of W^6+^ ions that migrate toward the titanium foil [14]. Therefore, with higher fluoride ion content, more W will be incorporated into the TiO_2_ nanotubes. The presence of C species is attributed to the ethylene glycol electrolyte, which is an organic electrolyte and can contribute to carbon doping onto TiO_2_ nanotubes [15,16].

**Table 2:** Energy-dispersive X-ray elemental analysis of WO_3_-loaded TiO_2_ nanotubes.

NH_4_F (wt %)	Atomic %			
	Ti	O	W	C
0	85.40	7.78	0.22	6.51
0.1	42.17	51.80	1.50	4.53
0.3	26.97	65.43	2.36	5.24
0.5	34.28	58.80	2.90	4.02

### 2.2. Phase Structure Analysis

**Figure 3:**
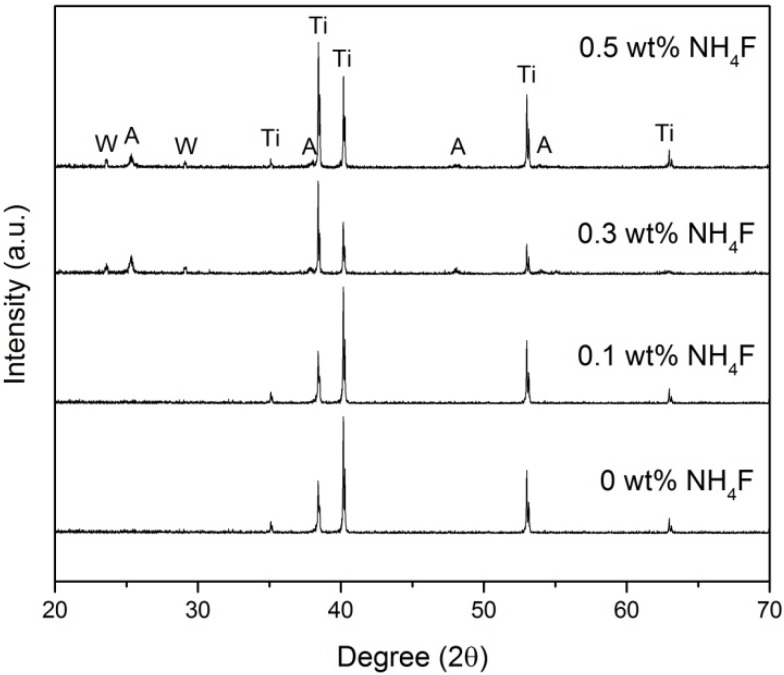
X-ray diffraction patterns of WO_3_-loaded TiO_2_ nanotubes produced with different fluoride ion content.

Figure 3 shows the XRD profile of the WO_3_-decorated TiO_2_ after annealing at 400 °C in air atmosphere for 4 h, which is the typical temperature and time for heat treatment to transform the amorphous structure of TiO_2_ into the crystalline anatase phase [17]. The XRD spectrum indicates the presence of the anatase phase of TiO_2_ (JCPDS No 21-1272). The diffraction peaks at 25.37°, 38.67°, 48.21°, and 54.10° are correspond to (101), (112), (200), and (105) crystal planes for the anatase phase, respectively. Furthermore, small additional peaks at 23.62° and 29.16° correspond with the (020) and (120) crystal planes of the monoclinic WO_3_ phase. We observed that the peaks corresponding to the anatase phase were most intense for the sample produced with 0.3 wt % NH_4_F, followed by 0.5 wt % NH_4_F and 0.1 wt % NH_4_F. Anatase peaks were not detected for the sample produced with 0 wt % NH_4_F. This corresponds with the increased growth of TiO_2_ nanotubes which can be explained with the faster movement of the Ti/TiO_2_ interface into the Ti metal due to the higher content of ionic species that move through the barrier layer at the bottom of the nanotube [18]. The improved pore deepening process results in longer nanotube length, which explains the increasing intensity of the anatase peaks. When fluoride ion content was further increased to 0.5 wt % NH_4_F, the intensity of the anatase peaks decreased slightly due to excessive chemical etching of the oxide layer during the chemical dissolution reactions which caused a decrease in nanotube length [8]. However, only the XRD pattern of sample produced with 0.3 and 0.5 wt % NH_4_F showed an obvious WO_3_ phase. A possible explanation would be that the XRD analysis was not sensitive enough to detect very low WO_3_ content within the TiO_2_ lattice of the samples produced using lower fluoride ion content due to the nearly similar ionic radius of W^6+^ and Ti^4+^ [19].

### 2.3. Raman Analysis

**Figure 4:**
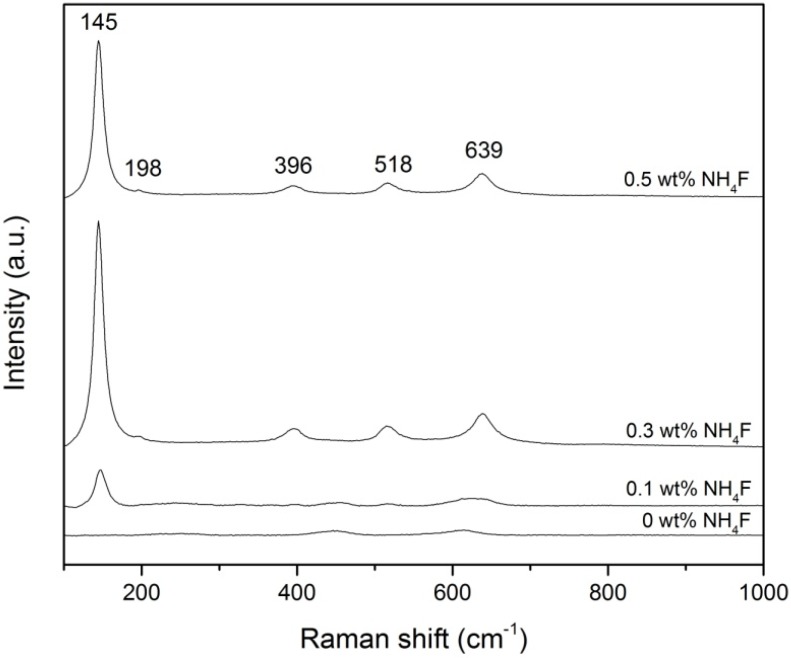
Raman spectrum of WO_3_-loaded TiO_2_ produced with different fluoride ion content.

Raman analysis was conducted to detect the presence of WO_3_ and to confirm the XRD inferences of WO_3_-loaded TiO_2_ nanotubes. Figure 4 shows the Raman spectrum obtained and there are five characteristic modes observed at 145, 198, 396, 518, and 639 cm^−1^. The mode at 145 cm^−1^ is strong and assigned as the E_g_ phonon of the anatase structure and the B_1g_ phonon of the rutile structure. The latter four modes are assigned as E_g_, B_1g_, B_1g_, and E_g_ modes of the anatase phase, respectively. The positions and intensities of the five Raman active modes correspond well with the anatase phase of TiO_2_ [20,21]. The lower intensity of anatase peaks for the 0 wt % and 0.1 wt % NH_4_F samples correspond with the short and small nanotubes produced. The sample produced with 0.3 wt % NH_4_F showed the highest intensity of anatase peaks because the TiO_2_ layer formed is denser and thicker. However, when fluoride ion content is increased to 0.5 wt %, the intensity of the anatase peaks decreased. This showed that more of the oxide layer had been dissoluted due to the higher chemical dissolution rate, leading to a thinner TiO_2_ layer [22]. Raman bands for WO_3_ were not detected because typical characteristic modes for WO_3_ are similar to those for anatase (e.g., 327, 714, and 804 cm^−1^) and were overlapped by bands for the anatase phase [17].

### 2.4. Optical Properties Analysis

**Figure 5:**
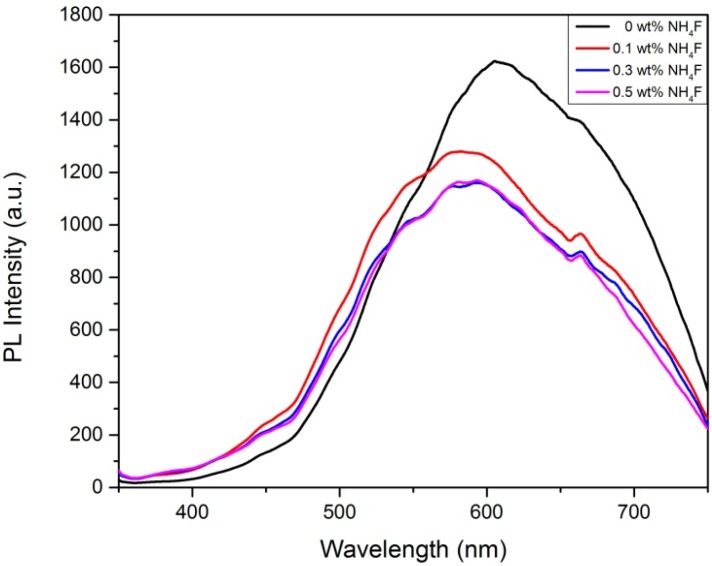
Smooth PL curve for WO_3_-TiO_2_ nanotubes produced with different fluoride ion content.

The PL (photoluminescence) emission spectra is a useful characterization tool that can be used to determine the efficiency of charge carrier trapping and to understand the fate of electrons and holes in the semiconductor because PL emission is the result of the recombination of free carriers [23,24]. Figure 5 shows the PL spectra of WO_3_-TiO_2_ nanotubes synthesized using different fluoride ion content. It can be clearly seen that the PL emission intensity for WO_3_-TiO_2_ nanotubes synthesized with 0 wt % NH_4_F is the highest. The sample produced using 0.3 wt % NH_4_F and 0.5 wt % NH_4_F showed the lowest and most similar PL emission intensities followed by the sample produced using 0.1 wt % NH_4_F. Since the PL emission mainly results from the recombination of excited electrons and holes, we can deduce that the lower PL intensity indicates that the WO_3_-TiO_2_ nanotubes synthesized using 0.3 wt % NH_4_F and 0.5 wt % NH_4_F have a lower recombination rate compared to the samples synthesized with 0 wt% NH_4_F and 0.1 wt % NH_4_F. The variation of PL emission intensity may be due to the presence of more WO_3_ species in the 0.3 wt % NH_4_F and 0.5 wt % NH_4_F samples, which suppressed the recombination of the photogenerated carriers and increased the charge separation of TiO_2_ [25]. This is due more to the cathodic valence and conduction band potentials of TiO_2_, which allows electron transfer from the conduction band of TiO_2_ down to the conduction band of WO_3_, thus suppressing the recombination of photogenerated carriers [26]. From the PL spectra, we are also able to determine the energy band gap of the WO_3_-TiO_2_ nanotubes for application purposes. The PL emission spectrum is useful in estimating the band gap energy (*E*_bg_) of the samples. The band gap energy (*E*_bg_) of the sample is calculated as follows: *E*_bg_ = *hc*/λ, where *E_bg_* is the band gap energy, *h* is Planck’s constant (4.135667 × 10^–15^ eV s), c is the velocity of light (2.997924 × 108 m/s), and λ is the wavelength (nm) of PL emission. In the photoluminescence spectra, the wavelength corresponding to the highest PL emission intensity is the light wavelength at which the sample is most active. By taking this wavelength value as λ, the energy band gap of the sample can be estimated. From the PL spectra, the samples show the highest emission intensity at wavelength of 580 nm, which corresponds with the energy band gap value of 2.14 eV. This band gap value is significantly lower than that of WO_3_ (2.8 eV) and TiO_2_ (3.2 eV), attributed to the presence of carbon species within the TiO_2_ nanotubes which significantly enhanced the visible light responsiveness of the WO_3_-loaded TiO_2_ nanotubes. The band gap energy of TiO_2_ is narrowed down by the mixing of the delocalized p state of the carbon species with the 2p orbital of the oxygen species in the valence band of TiO_2_, which shifts the valence band edge of TiO_2_ upwards.

### 2.5. Mercury Removal

**Figure 6:**
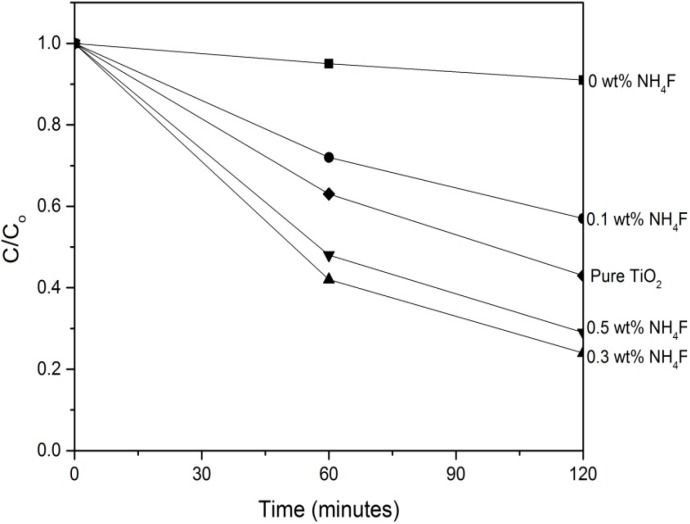
Removal of Hg^2+^ by WO_3_-TiO_2_ nanotubes and pure TiO_2_ nanotubes.

**Figure 7:**
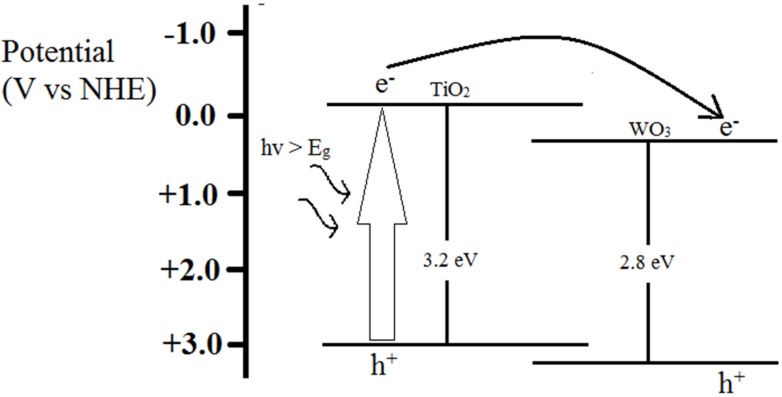
Suppression of recombination of photogenerated carriers due to coupling of WO_3_ and TiO_2_.

The photocatalytic reduction of Hg^2+^ in aqueous solution by WO_3_-TiO_2_ nanotubes under UV illumination was investigated. Before photocatalytic reactions, the adsorption of mercury on the catalyst was allowed to be established in the dark in order to distinguish between photocatalytic Hg^2+^ uptake and the removal of Hg^2+^ by adsorption. As shown in Figure 6, the nanotubes produced using 0.3 wt % NH_4_F showed the best mercury removal performance where 76% of the mercury in the initial solution was removed after two hours of UV irradiation. The sample produced using 0.5 wt % NH_4_F showed a slightly lower performance where 71% of the mercury in the initial solution was successfully removed. The samples produced with 0 and 0.1 wt % of NH_4_F showed the lowest performance of mercury removal where only 9% and 43% of the initial mercury concentration were successfully removed, respectively. The reason for the better performance of samples produced with 0.3 wt % NH_4_F is due to the larger active surface area which enables more Hg^2+^ to adsorb onto the catalyst surface to undergo reduction to Hg^0^ [27]. Furthermore, the larger active surface area generates more photo-induced electron-hole pairs which allow more Hg^2+^ to undergo reduction [28,29]. In order to compare the photocatalytic activity of WO_3_-TiO_2_ nanotubes with pure TiO_2_ nanotubes, pure TiO_2_ nanotubes were produced using the same parameters as the WO_3_-TiO_2_ nanotubes synthesized using 0.3 wt % NH_4_F except replacing the tungsten cathode with a platinum cathode. As compared to WO_3_-TiO_2_ nanotubes, pure TiO_2_ nanotube arrays showed a lower efficiency of mercury removal, where 57% of the mercury in the initial solution was removed after two hours. This shows that the coupling of WO_3_ and TiO_2_ gives a significant improvement in the photocatalytic activity of the nanotube arrays due to the suppression of the recombination of the photogenerated carriers and the increased charge separation of TiO_2_. As illustrated in Figure 7, when a photon with not enough energy to excite TiO_2_ but with enough energy to excite WO_3_ is incident, the hole that is created in the WO_3_ valence band is excited to the conduction band of TiO_2_, while the electron is transferred to the conduction band of TiO_2_. This electron transfer increases the charge separation, thus leading to a lower recombination rate [25]. Furthermore, since the valence and conduction band potentials of TiO_2_ are more cathodic than that of WO_3_, photogenerated electrons can transfer from the conduction band of TiO_2_ down to the conduction band of WO_3_. This will suppress the recombination of photogenerated carriers [26].

## 3. Experimental Section

**Figure 8:**
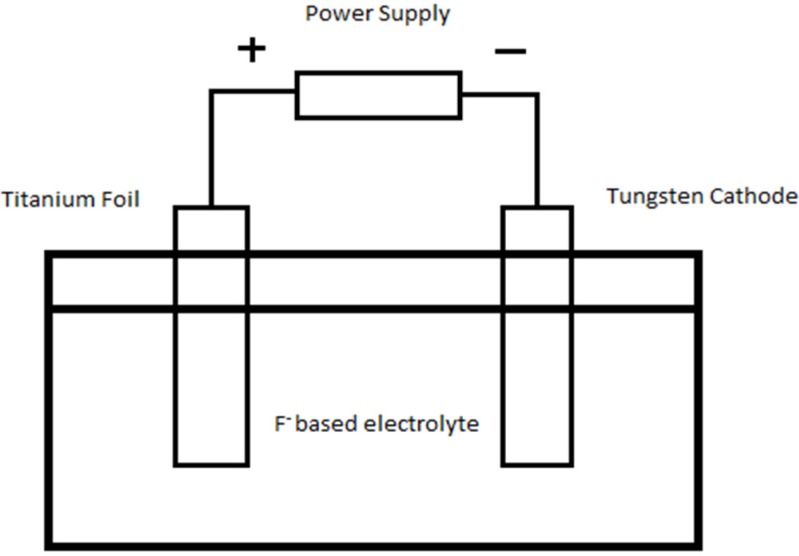
Schematic drawing of an electrochemical cell in which the Ti electrode is anodized.

The experiments were carried out in a two-electrode electrochemical cell as shown in Figure 8, where the two electrodes were placed 2 cm apart. Titanium (Ti) foil (0.127 mm, purity 99.6%, Sigma Aldrich, St. Louis, MO, USA) (5 cm × 1 cm dimension), over which WO_3_-loaded TiO_2_ nanotubes were grown, was used as the anode while tungsten foil (0.127 mm, purity 99.9%, Sigma Aldrich, St. Louis, MO, USA) was the counter electrode. The electrolytes were ethylene glycol (EG, Friendemann Schmidt, Germany) with dissolved hydrogen peroxide (H_2_O_2_, Friendemann Schmidt, Germany) and ammonium fluoride (NH_4_F, Merck, Kenilworth, NJ, USA) in amounts ranging from 0 wt % to 0.5 wt %. H_2_O_2_ functions as oxygen provider for the higher oxidation rate of Ti to form TiO_2_ nanotubes at a rapid rate. Anodization was carried out at voltage of 40 V for a duration of 60 min. As-anodized anodic WO_3_-loaded TiO_2_ samples were cleaned using deionized water followed by sonication in acetone (Friendemann Schmidt, Germany) to remove the remaining occluded ions from the anodized solutions or barrier oxide layer. The samples were then subjected to calcination at 400 °C for 4 h in air atmosphere.

The morphologies of anodic WO_3_-loaded TiO_2_ nanostructures were observed by field emission scanning electron microscopy (FESEM, FEI Quanta 200F Environmental SEM with EDAX, FEI, Hillsboro, OR, USA) microanalysis at 5 kV. The structural variation measurements and phase determinations were done using X-ray diffraction (XRD, Bruker D8 Advance diffractometer, Bruker Corporation, Billerica, MA, USA) analysis conducted from 10 to 80° with Cu Kα radiation (α = 1.5406 Å). The phase composition was determined using Raman Spectroscopy (Renishaw in Via, Renishaw plc, Gloucestershire, UK) with a 514.5 nm Ar^+^ laser as an excitation source.

**Figure 9:**
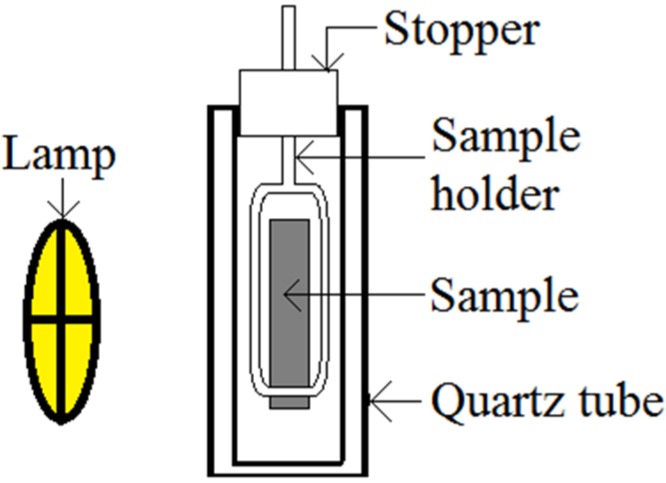
Schematic diagram of the photocatalytic reactor in which photocatalytic degradation was performed.

The performance of the WO_3_-decorated TiO_2_ nanotube arrays for mercury removal was studied by dipping annealed samples in 100 ml of 100 ppb mercury chloride (HgCl_2_) solution in a photoreactor consisting of quartz glass, as shown in Figure 9. After leaving the samples in the reactor for 30 min in dark environment for dark adsorption, the samples were photoirradiated at room temperature by using TUV 96W UV-B Germicidal light. Then, 5 mL solution was withdrawn from quartz tubes every 60 min. A mercury analyzer (NIC RA-3120, Nippon Instruments Corporation, Osaka, Japan) was used to measure the concentration of the HgCl_2_ solution.

## 4. Conclusions

In this study, the effect of fluoride ion content on the formation of WO_3_-TiO_2_ nanotubes was investigated. WO_3_-TiO_2_ nanostructures were successfully produced using 0.3 and 0.5 wt % NH_4_F. In order to grow well-aligned WO_3_-decorated TiO_2_ nanotubes, the fluoride ion content was optimized at 0.3 wt % NH_4_F for 60 min of anodization duration at a potential of 40 V. Lower fluoride ion content (0.1 wt %) provides a low dissolution rate of the oxide layer due to insufficient fluoride ions whereas excess fluoride ions (0.5 wt %) cause an increased pore diameter and reduced nanotube length due to excessive chemical etching of the oxide layer. Furthermore, WO_3_-decorated TiO_2_ nanotubes synthesized with 0.3 wt % NH_4_F showed the best mercury removal ability due to the larger active surface area that generated more photo-induced electron-hole pairs, better charge separation, and less charge carrier recombination.
